# Supplementary material for: Associations between probable REM sleep behavior disorder, olfactory disturbance, and clinical symptoms in Parkinson’s disease: A multicenter cross-sectional study
Source: PLoS One. 2021 Feb 19;16(2):e0247443. doi: 10.1371/journal.pone.0247443 (PMC7894886; doi:10.1371/journal.pone.0247443)
Supplement: Attachment — Submitted filename: Response to Reviewers.docx [file pone.0247443.s001.docx]

Responses to the Comments of Reviewer #1

We thank the Reviewer for valuable comments.

Page 11, line 167-169: The authors claim tha disease duration was significantly longer in PRBD both on RDBDSQ-J and RBDQ1 ("these results were the same as those based on the RBD1Q.") This does not match the results in table 2, p-values 0.03 and 0.11, respectively.

***Response:*** We thank the Reviewer for this valuable comment. The Reviewer is correct.

We have revised and added text as follows.

“These results were the same as those based on the RBD1Q excluding disease duration, HY scales and MMSE.” in the Results (Line 173-174)

.

Page 12, results section: Line 180-186: Describe the statistical method (logistic regression)

***Response:*** We described the statistical method.

….by logistic regression analysis,…, (Line 187-188)

Page 13, Discussion: Line 205-207: The sentence is repeated from the abstract and the results section, but not discussed. Do you have any commets on the difference? Are there any particular items on the RBDSQ that apply on those who are negative on RBD1Q? For instance those items that concerns motor activity t night

***Response:*** Regarding the agreement of the two questions, when we evaluated the agreement of the two questions by the Cohen’s Kappa coefficient, it showed a good agreement of 0.62. (Line 160-161 in Result)

We have added Figure 2 (comparison of characteristics between PD patients diagnosed pRBD only by RBDSQ-J and RBD1Q).

We devised as follows.

“The differences of clinical features between these two groups were HY scale, sleep and fatigue problems, which scores increased in patients with pRBD diagnosed only by the RBDSQ-J. This result suggested that patients with severe sleep problems and fatigue affected to diagnose pRBD by RBDSQ.” (Line 212-215)

In general, the language is clear and easily understandable. Some parts, particularly in the discussion, are however somewhat difficult to understand. Furthermore, several sentences should be rephrased.

***Response:*** We would like to thank you for your detailed suggestions. We have revised the text according to your suggestions.

Some suggestions:

Page 3:Abstract, Methods, line 43: The participants were 404 non-demented PD patients

　We changed text. (Line 43 in Abstract)

Page 7, Methods, line 99-100: Eighteen patients were excluded due to a score <24 on the MMSE and one patient was excluded due to insufficient information.

　We have changed text. (Line 101-102 in Methods)

Page 7, line 103: “multicenter hospitals in Japan” – needs to be rephrased. How many hospitals?

We have added text …” seven hospitals” (Line 105, in Methods)

Page 8, line 117: Both odor identification tests… (not the both)

We have corrected this sentence.

Page 13, line 205: This is the first study to describe..

We deleted this sentence because our study was not first study to describe. (Added ref. 29 which was the first study to compare two RBD screening questions)

Page 16, line 254: sentence should be rephrased

We deleted this sentence because it was confusing.

Page 17, line 268-269: obvious cognitive decline, not cognitive function

We have corrected this sentence.

Responses to the Comments of Reviewer #2:

We thank the Reviewer for valuable comments.

1. The authors should retitle the manuscript using "probable RBD" as vPSG is mandatory for the definite diagnosis of RBD and the authors used screening questionnaires instead.

***Response:*** We changed the title to "probable RBD".

2. The authors used two widely used screening questionnaire to investigate RBD in their population, namely the Japanese version of RBDSQ and the RBD1Q. The latter has been validated in a cohort of isolated RBD, finding 84.2% of sensitivity and 96.2% of specificity. Recently, RBD1Q has been used in a large cohort of 97 non-demented PD patients, showing 67.7% of sensitivity, 82.9% of specificity, with 87.5% of PPV and 59.2% of NPV when administered prior to any kind of clinical interview. (Figorilli et al. doi: 10.1093/sleep/zsz323).

***Response:*** We would like to thank you for introducing us the valuable literature.

We have added this literature in References 29, and added contents in the introduction and discussion.

“Recently, the RBD1Q showed 67.7% of sensitivity, 82.9% of specificity, with 87.5% of positive predictive value (PPV) and 59.2% of negative predictive value (NPV) [29].” (Line 86-88 in Introduction)

3. How do the authors explain the differences between the two questionnaires in terms of associations with motor and non-motor symptoms?

***Response:*** Regarding the agreement of the two questions, when we evaluated the agreement of the two questions by the Cohen’s Kappa coefficient, it showed a good agreement of 0.62. (Line 160-161 in Results)

We have added Figure 2 (comparison of patients diagnosed with RBD only in each question).

I devised as follows.

“The differences of clinical features between these two groups were HY scale, sleep and fatigue problems, which scores increased in patients with pRBD only diagnosed by the RBDSQ-J. This result suggested that patients with severe sleep problems and fatigue affected to diagnose pRBD by RBDSQ.” (Line 212-215 in Discussion)
