# Supplementary material for: Associations between probable REM sleep behavior disorder, olfactory disturbance, and clinical symptoms in Parkinson’s disease: A multicenter cross-sectional study
Source: PLoS One. 2021 Feb 19;16(2):e0247443. doi: 10.1371/journal.pone.0247443 (PMC7894886; doi:10.1371/journal.pone.0247443)
Supplement: Attachment — Submitted filename: ResponseTo Reviewers2.docx [file pone.0247443.s002.docx]

Reviewer #1: The revised manuscript is improved. However, I still have some comments.

***Response***: We thank the Reviewer for this valuable comment. We have revised our paper according your mentions. A native speaker has proofread our paper.

1: The term probable RBD should be introduced earlier in the manuscript, not in the statistics section. I would suggest that you introduce the term pRBD in line 89, because you aim to investigate the prevalence of pRBD, not RBD.

2: The term pRBD should be used consistently throughout the manuscript when you refer to your present study, for instance in line 113.

***Response:*** We thank the Reviewer for this valuable comment. We have revised this as “probable RBD” (lines 83, 85, and 108).

3: Line 160: Sentence should be rephrased.

***Response:*** We revised the sentence as follows (line 157-158):

“The two questionnaires showed moderate agreement, with a Cohen’s kappa coefficient of 0.62.”

4: Line 210: the study showed good agreement (not the good agreement)

***Response:*** We revised the sentence as follows (line 209):

“This study showed the moderate agreement between the RBDSQ-J and RBD1Q.”

5: Line 212 and table 2: Significantly more men diagnosed by RBDSQ-J. Any comments?

***Response:*** We added the following comments in the Discussion (lines 215–217):

“Since it has been confirmed that the proportion of males is high among patients with RBD, pRBD evaluation by the RBD1Q can be considered to reflect gender differences more accurately.”

6: Line 214/215: Sentence is not easily understandable and should be rephrased.

***Response:*** We revised this sentence, as suggested (lines 209–217).

“This study found moderate agreement between the RBDSQ-J and RBD1Q, …gender differences more accurately.”

7: Line 224: As a result, a significant difference…. Should be rephrased, “A significant difference..”

***Response:*** We revised this sentence, as suggested (line 226).

Reviewer #2: The authors adequately addressed all the points raised by the reviewer.

However, the English should be revised.

A native speaker has proofread our paper.

We have revised our paper according Reviewer #1 comments.
